# Improving the efficacy and effectiveness of evidence-based psychosocial interventions for attention-deficit/hyperactivity disorder (ADHD) in children and adolescents

**DOI:** 10.1038/s41398-024-02890-3

**Published:** 2024-06-08

**Authors:** Anil Chacko, Brittany M. Merrill, Michael J. Kofler, Gregory A. Fabiano

**Affiliations:** 1 New York University, New York, NY USA; 2 Florida International University, Miami, FL USA; 3 Florida State University, Tallahassee, FL 32306 USA

**Keywords:** Human behaviour, ADHD

## Abstract

Attention-deficit/hyperactivity disorder (ADHD) is a prevalent, chronic, and impairing mental health disorder of childhood. Decades of empirical research has established a strong evidence-based intervention armamentarium for ADHD; however, limitations exist in regards to efficacy and effectiveness of these interventions. We provide an overview of select evidence-based interventions for children and adolescents, highlighting potential approaches to further improving the efficacy and effectiveness of these interventions. We conclude with broader recommendations for interventions, including considerations to moderators and under-explored intervention target areas as well as avenues to improve access and availability of evidence-based interventions through leveraging underutilized workforces and leveraging technology.

## Evidence-based treatments for ADHD - an Overview

Multiple groups, committees, and professional organizations have provided the field with recommendations for evidence-based treatment approaches for ADHD. There is clear consensus across these recommendations that pharmacological treatments, notably stimulant medication, psychosocial treatments, and a combination of these two approaches have the strongest evidence base. Table 1 provides a brief overview of the major conclusions of each guideline for the treatment of ADHD in children. It is clearly recommended that families should receive psychoeducation regarding ADHD, and that the evidence-based psychosocial treatments are behavioral parent training (BPT), behavioral interventions in classroom and peer settings, and organizational skills training [1–5].

**Table 1:** Review of practice parameters and treatment guidelines for ADHD.

Organization	Overall recommendation for ADHD treatment	Specific notes and recommendations
American Academy of Child and Adolescent Psychiatry [6]	• “A well thought out and comprehensive treatment plan should be developed… should include parental and child psychoeducation about ADHD and its various treatment options (medication and behavior therapy), linkage with community supports, and additional school resources” (pp. 902) • “Initial psychopharmacological treatment of ADHD should be with an agent approved by the Food and Drug Administration” (pp. 904) • “If none of the above agents result in satisfactory treatment … then consider behavior therapy and/or the use of medications not approved by the FDA for the treatment of ADHD” (pp. 907)	• “If a patient with ADHD has a robust response to psychopharmacological treatment … Then psychopharmacological treatment of the ADHD alone is satisfactory” (pp. 912)
American Academy of Pediatrics [20]	• Preschool: “Evidence-based behavior PTBM and/or behavioral classroom interventions as the first line” (pp. 10) • Elementary and middle school: “Prescribe US FDA-approved medications for ADHD, along with PTBM and/or behavioral classroom intervention (pp. 11) • Adolescents: “prescribe FDA-approved medications for ADHD with the adolescent’s assent … encouraged to prescribe evidence-based training interventions and/or behavioral interventions” (pp. 11)	• Recommendations vary based on the child’s age: under six, six to 12, and 12–18.
Coghill [124]	• “Clinicians should be confident ADHD medications are effective, well-tolerated, and safe. Parent training provides an important complement” (pp. 19) • “ADHD-focused parent training remains the first-line intervention for preschool children” (pp. 15)	• Integrated recommendations from six different practice guidelines.
Society for Clinical Child and Adolescent Psychology [1–4]	• Behavioral parent training, contingency management in classroom settings, behavioral peer interventions, and training interventions in organizational skills meet criteria for well-established treatments.	• Medication was not explicitly reviewed; focus was on psychosocial treatments
Society for Developmental and Behavioral Pediatrics [5]	• “Evidence-based behavioral and educational interventions are foundational for the treatment of complex and ADHD and should be implemented at the outset of treatment” (pp. S43) • It is often necessary to combine these approaches with pharmacological treatments” (pp. S36)	• Guidelines emphasize that focus of treatment should be on functional outcomes, rather than ADHD symptoms.

*ADHD* attention deficit/hyperactivity disorder, *FDA* food and Drug Administration, *PTBM* parent training behavior management.

There are inconsistencies among the guidelines that make broad statements of consensus difficult. For instance, there are differences in precision in recommendations for psychosocial treatments, with some very broad in scope [6] compared to others with more precise recommendations regarding particular treatment types (e.g., BPT [2]) and particular populations (e.g., children under six; [5]). Broad suggestions of seeking “psychological” or “educational” treatment is unhelpful in some guidelines and practice parameters, as there are many approaches that fall under this category and some are clearly efficacious whereas other approaches commonly deployed do not have evidence of efficacy for ADHD [1–4]. There are also differences in the strength of recommendations, with more contemporary guidelines emphasizing multimodal treatments more so than older guidelines. However, perhaps most notably, there is not clear consensus among the recommendations on the best sequence or combination of treatments for ADHD, even though this is a key question for most families pursuing treatment for ADHD. It is also important to note that most guidelines focus on proximal ADHD treatment – as ADHD is now conceptualized as a life-course persistent disorder [7], treatment efforts will need to be protracted across time and appropriate for evolving developmental levels.

## Efforts at improving efficacy and effectiveness of psychosocial intervention for ADHD: what do we know and where do we go?

Given the prominent role of psychosocial, primarily behavioral interventions, for ADHD, we highlight the evidence for several of these key interventions, integrating the literature on improving efficacy and effectiveness of these interventions. We also discuss digital therapeutics given the explosion in its availability and purported efficacy for children with ADHD. Following this, we close with potential broad future directions for psychosocial treatments for children with ADHD.

### Behavioral parent training

Behavioral parent training (BPT) is likely the most well-studied psychosocial intervention for children’s mental health disorders, including for ADHD [8]. It serves as the first line intervention approach for younger children with ADHD and is an integral part of comprehensive intervention approaches for school-age children with ADHD. Importantly, BPT is less studied in adolescents with ADHD. Although parenting is not etiological to ADHD, there are clear reasons to focus on parenting when supporting a child with ADHD. Of primary importance is that raising a child with ADHD is stressful, and not surprisingly, elicits ineffectual parenting practices (e.g., inconsistent, harsh, lax, overreactive, less responsive). As a result, parents often have lower parenting efficacy/competence, higher levels of coercive management practices, utilize maladaptive coping strategies (e.g., increased use of alcohol), and have more negative attributions/perceptions of their child [8]. These parent-level challenges can be addressed, in part, by supporting parents to utilize more proactive and effective parenting practices which can help improve functioning for themselves and ultimately their children. Importantly, the most common comorbidities with ADHD, Oppositional Defiant Disorder [ODD] and Conduct Disorder (CD) are best treated with BPT—making BPT an essential treatment for the most common disruptive behavior disorders in childhood [8].

BPT is based on operant-conditioning and social learning theories, with techniques that focus on antecedents (e.g., effective instructions, rules) and consequences (e.g., active ignoring, time-out from positive reinforcement) of behaviors. This core content is delivered in a flexible manner with varying formats (e.g., group, individual) durations (brief vs longer), with or without child involvement, delivery (e.g., with or without video-based learning). Moreover, over the past two decades, there have been efforts at tailoring BPT to meet the needs of specific populations (e.g., single mothers, fathers; Latine; [9–12]). These BPT programs, often referred to as “homegrown” BPT as compared to commercialized BPT programs (those that have been more extensively developed, manualized and are commercially available; e.g., Defiant Children [13]) retain the core content of traditional BPT but have modifications to format or additional content that are based on the needs of the targeted populations. Overall, commercialized BPT and homegrown BPT have been found to be effective in improving the functioning of children with ADHD and their parents [14–16]. A recent meta-analysis also suggests sustained benefits of BPT over the course of a year on child ADHD symptoms, parenting behavior, parenting sense of competence and parental mental health [17]. The significance of BPT should be, however, put into a broader context to appreciate the clinical benefits of this intervention. While multiple randomized controlled trials have established the statistical significance of BPT for ADHD, the effect size for BPT ranges from small to medium effects, depending upon the outcome [18]. This means that for many outcomes, the effect sizes would be “visible to the naked eye of a careful observer” [19]. While there is limited data, only a significant minority of children are “normalized” following BPT [15]. Collectively, a more nuanced perspective on BPT for ADHD suggests that it is an evidence-based intervention that can result in visible improvements on key outcomes. There is room, however, to improve the potency of BPT. The implications of the findings reported above suggest several broad areas for further investigation. First is to increase access to BPT given the benefits of the intervention. Wolraich et al. [20] note that there is a lack of an adequate pool of behavioral and mental health specialists who are available to provide evidence-based psychosocial treatments for ADHD, including BPT. National data suggest that the majority of youth with ADHD are receiving no treatment, even when identified, and the lack of BPT treatment is most pronounced in young children with ADHD [21]. Efforts at utilizing technology to increase the workforce offers novel and promising approaches to address this issue [22].

A second area is to increase the potency of BPT. We believe there are multiple ways to achieve this goal, with the most apparent being improving the extent to which parents fully engage in BPT, given the relation between increased engagement and improved potency of outcomes [23]. It is common for families of children with ADHD, even those who have enrolled in BPT, to not initiate treatment or drop out of BPT prior to completion [9, 23]. Given this, there have been notable efforts at improving engagement to BPT through addressing perceptual (e.g., expectations about BPT), practical (e.g., transportation) and cultural barriers to treatment prior to BPT [10, 24] as well as during BPT [25]. Given that engagement challenges often involve practical barriers (e.g., transportation, child care, fixed appointment times), there has been efforts at increasing access through reducing these barriers such as providing BPT through mobile applications [26], web-based platforms [27] and telehealth delivery [28, 29]. These efforts have led to improved engagement and associated outcomes for families, beyond traditional BPT [30]. Engagement with BPT remains an important area of research, particularly the extent to which these enhancements to BPT can be readily applied in routine settings [25, 31, 32], an understudied empirical question.

A second and meaningful line of research to improve the potency of BPT has been focused on improving specificity of BPT content by translating contemporary theories of ADHD into refinements to BPT. Van der Oord and Tripp [33]), utilizing contemporary motivational reinforcement-based theories of ADHD, suggest that given altered reinforcement sensitivity in ADHD, rewards and punishment should be judiciously provided. As an example, they note evidence that while mild negative punishment (e.g., response-cost, time-out from positive reinforcement) improves on-task behavior in children with ADHD, mild punishment can also lead to more errors on tasks, increased emotionality in children with ADHD, missed learning opportunities, and lack of task persistence [34, 35]. These authors note caution in the use of punishment, especially positive punishment (e.g., verbal reprimands), with children with ADHD. Rather, there should be a focus on rewarding alternative adaptive behaviors to reduce the need to use punishment. These theory-driven considerations to adapting BPT are important, yet empirically understudied. As such, the extent to which these ADHD-theory-adapted BPT results in improved outcomes relative to standard BPT is not known. Importantly, however, some efforts in this area have resulted in little difference for ADHD-adapted BPT relative to standard BPT. As an example, in an RCT, the New Forest Parenting Program (NFPP), which was developed to address underlying mechanisms of ADHD (self-regulatory and cognitive problems [36] was found to be no better than a standard BPT program and in some areas less effective (e.g., parental stress, parenting behavior, parent reports of ADHD symptoms at follow-up) for preschool children with ADHD [37]. These data suggest the importance of rigorously evaluating novel approaches that are considered improvements to BPT to well-established traditional BPT for ADHD.

Overall, the efficacy of BPT suggests that this should be first-line intervention approach for children with ADHD, with significant and noticeable effects of BPT on both parent- and child-level outcomes with maintenance of gains over the course of a year. This statement comes with the caveat that there is room for improvement in the potency of BPT. As we will discuss in the future directions section below, greater attention must be given to dissemination of BPT (and all psychosocial interventions for ADHD) within routine systems of care and evaluation of the effectiveness of these interventions alone and in combination when delivered within these systems.

### Behavioral classroom management

ADHD is largely defined by challenges in settings such as schools where behavioral expectations are often demanding of attention capacity and self-control, and so it is not surprising that many of the efficacious treatments for ADHD have focused on improving academic functioning and classroom behaviors. Children with ADHD are effectively treated with classroom contingency management strategies [38]. Systematic reviews [1, 2, 4, 5, 39] as well as meta-analyses [40–42] clearly illustrate that behavioral classroom management is an efficacious treatment for ADHD.

As noted above, behavioral classroom management is an efficacious treatment for ADHD. Before discussing evidence for efficacy and effectiveness of behavior classroom management, it is worth noting approaches that do not strongly support positive outcomes. In the United States, children with ADHD are eligible for behavioral classroom management support through Section 504 Accommodation plans administered through the Americans with Disabilities Act or through an Individualized Education Program if the committee on special education determines it is needed. These policies have provided school-based behavioral supports for students with ADHD for over 30 years. However, given that follow-up studies indicate that the long-term educational outcomes for students with ADHD are modest, at best [43–46], it is important to emphasize that these accommodation plans or individualized education programs are only useful if they include effective interventions and supports for the child or adolescent with ADHD.

Practically speaking, behavioral classroom management approaches that are effective will include setting clear goals and rules, ensure that the child receives clear feedback on progress toward meeting goals, and that consequences, typically rewards and privileges contingent on meeting behavioral goals and following rules, are provided liberally. It is important to note that positive behavior support strategies are interwoven into the fabric of elementary school classrooms - teachers provide rules and structure for activities, there is praise issued for appropriate behavior, and schools have standard discipline procedures including office referrals or detentions for rule violations. Feedback is provided on a regular, if infrequent basis (i.e., on quarterly report cards). For most children with ADHD this provides a reasonable baseline of behavioral classroom management, but additional strategies and supports are typically needed to make the overall approach to supporting a child with ADHD more efficacious.

Children with ADHD typically need much more frequent behavioral feedback and positive consequences for appropriate behavior in schools. For that reason, a daily report card is among the most efficacious positive behavior supports within a classroom [47, 48]. The daily report card has long been used effectively to treat ADHD, monitor outcomes, and open a daily line of communication between teachers and the child’s parent [49–57], and it is a procedure aligned with a long tradition of using contingency management with children with disruptive behavior in general educational settings [58] and in special education settings [57, 59, 60]. In addition to being among the most efficacious classroom interventions, it is also one of the most cost effective [61].

Recent changes in the ways schools address social, emotional, and behavioral challenges may promote greater effectiveness of the implementation of classroom behavior management strategies. Multi-tiered systems of support (MTSS [62] in schools conceptualize the behavior of children as being within a continuum, and through regular screening and progress monitoring, provide more intensive intervention, when indicated and for as long as is needed. Currently MTSS efforts in schools are focused on academic achievement targets, and there is less emphasis on MTSS for behavior [63]. However, the MTSS model of screening and intervention is similar to the single case design approach to intervention that has been long-used within the ADHD treatment literature [4, 5, 47]. In this approach, following the collection of baseline data, behavior classroom management interventions are systematically introduced to evaluate their effectiveness. Educators also benefit from ongoing coaching, support, and monitoring of progress to promote consistent and protracted use of these behavioral interventions [64].

Supporting this approach to school-based intervention is a recent study that evaluated the effectiveness of different sequences of ADHD treatment [65]. In this study, using a sequential, multiple assignment randomized trial (SMART [66]), children were randomly assigned to begin the school year with one of the two evidence-based treatments for ADHD - a low dose of stimulant medication or an initial course of behavior therapy (eight parent training sessions and a daily report card intervention at school). Teachers provided feedback on how the child was functioning in the classroom, and if there was evidence of impaired functioning, the child was then randomly assigned to more treatment – either a greater dose of the treatment at the start of the school year, or a other modality. Thus, children could have a treatment sequence of: (1) medication followed by an increased dose of medication; (2) medication with behavior therapy added; (3) behavior therapy followed by an increased dose of behavior therapy; or (4) behavior therapy followed by medication. Results were interesting as they illustrated the best sequence of treatment for reducing discipline referrals and disruptive behaviors observed in the classroom were those that started with behavior therapy first. Further, the behavior therapy first approaches also cost less to implement across the school year than the treatment sequences that included medication [61]. Importantly, this study of the effectiveness of treatment sequencing spanned an entire school year, improving upon the research base of efficacy treatments where many studies focused on shorter time periods. While the Pelham study provides a foundation for considering combined and sequenced approaches, far less has been done on the effectiveness of behavioral classroom approaches when conducted within and supported entirely by school-staff over the course of multiple school years.

Overall, there is strong support for behavioral classroom interventions, including the Daily Report Card [48, 50, 67], and it is strongly recommended that this intervention be initiated for children with ADHD experiencing classroom-based impairment. For older children (e.g., middle school, high school), a behavioral contract may be used to initiated contingency management across the multiple classrooms characteristic of this grade level. Educators and parents should ensure that school-based interventions are implemented consistency and continuously, as school-based behavioral challenges are likely to extend across school years and grade levels.

### Organization skills training

Related to their difficulties staying on task and following the rules in the classroom setting, children with ADHD have impaired organization, time management, and planning skills that undermine their academic abilities and potential. Homework management and organizational skills have been shown to predict concurrent GPA and later academic outcomes [68, 69]. Organizational skills training (OST) interventions utilize behavioral methods to teach skills directly to students with ADHD. The training programs often include behavioral management procedures administered by a counselor, parent, or teacher to reinforce skill use and progress in treatment. Organizational interventions have largely targeted middle school to early high school students with ADHD (ages 10–14 [70]), with sessions focusing on materials organization, understanding time and time management, and planning larger assignments. Session frequency and length vary widely from about 10, 60-minute family sessions in a clinic [71] to 40, 2.5-hour student sessions in an after-school setting [72]. Multicomponent OST packages lead to improvements in organizational skills, planner use, and adolescent impairment [73].

Embedding OST in schools is key to enhancing the reach of these interventions. Though availability of school personnel to implement OST varies across districts, current work aims to train school counselors to implement OST with students with ADHD [74]. Langberg and colleagues [74] found that OST delivered by school staff led to improvements in organization, time management, and planning skills as performance and behavior during homework based on parent-report. Importantly, these results were found despite the school counselors receiving only 2 h of training in the intervention with no ongoing supervision. Purposefully limiting training and post-training investment allows for the examination of treatment effects in the context in which they would likely be provided—i.e., in schools, by school mental health providers with little funding for training and ongoing supervision. Toward the same goal of increasing the sustainability of OST within schools, investigators are developing online tools to assist school staff with OST implementation at low to no cost [75].

Psychosocial treatments for children with ADHD have primarily been researched in elementary-age youth, and early work in developing organizational skills interventions addressed this gap by upwardly extending treatments to middle school age youth. As the evidence accumulated that such interventions were efficacious among children with ADHD in middle school [1, 2], further developmental extension was clearly justified [76]. adapted their OST program developed for middle schoolers to pilot with high school students with ADHD. Pilot data demonstrated feasibility and indicated that high schoolers may need about 50 sessions to benefit from the OST program. In the full-scale randomized clinical trial [77], high schoolers attended an average of 40 brief OST sessions while their caregivers attended an average of 4 behavioral parent training sessions. Compared to the control group, beneficial effects of treatment were found on parent-reported academic functioning and organizational skills, and no significant effects on grades, teacher-reported, or self-reported outcomes were found. Results are promising, and much more work is needed to support the academic functioning of older adolescents with ADHD.

### Digital therapeutics

Of all the available non-medication treatments, this category is the only one that features protocols patented by the US Patent and Trademark Office (USPTO) and cleared by the U.S. Food and Drug Administration (FDA) for the treatment of ADHD. It is also the most controversial, with competing consensus statements concluding that these types of interventions have proven effective vs. proven ineffective [78], millions of dollars in fines for false advertising [78], and a perceived disconnect between benchmarks for effectiveness between the FDA and professional organizations that evaluate psychosocial treatments for children [79]. Overall, there appears to be evidence for small benefits of cognitive training on reducing inattentive symptoms, and potentially overall ADHD symptoms [80]. However, we contend that omnibus meta-analytic effect sizes are fundamentally uninterpretable in the context of cognitive training/digital therapeutics because the treatments that fall under this general umbrella feature wide variations in neurocognitive/neurological training targets, conceptual models of neurocognition that define these intended training targets, success/failure to meaningfully engage and improve the intended training target(s), and technologies employed to ‘hit’ the intended target(s). Therefore, what these treatments share are more peripheral features, rather than core mechanistic features necessary to meet meta-analytic assumptions. Even interventions given the same, more specific label (e.g., “working memory training”) vary widely in their approach, conceptual basis, and success/failure at engaging their mechanistic target as shown previously [81]. It will be important for the field to evaluate each intervention on its own merits because these protocols generally share very little with each other except for the use of a serious games approach [82] to engaging children in treatment.

Several neurocognitive training protocols have been developed and tested for children with ADHD; we briefly review three of the most prominent: EndeavorRX, CogMed, and Central Executive Training (Cenextra; an intervention developed by one of the co-authors). All three of these approaches are computerized digital therapeutics that include gaming elements and adaptive changes in difficulty. *EndeavorRX* trains cognitive attention abilities [83], *Cenextra* trains the ‘working’ components of working memory [84] and *CogMed* is intended to improve attention and working memory abilities [85], though clinical trial and meta-analytic evidence indicates that CogMed successfully engages short-term memory but not working memory abilities [81, 86, 87].

In terms of efficacy, *EndeavorRX* showed early promise in pilot/uncontrolled studies given generally favorable feasibility, acceptability, and engagement data [83]. In addition, EndeavorRX showed potential for reductions in parent-reported ADHD symptoms in proof-of-concept and open-label trials with children with ADHD [88, 89]. However, the only controlled trial to date [83] demonstrated that these potential reductions were likely attributable to placebo effects – that is, Endeavor RX failed to show superior improvements in ADHD behavioral symptoms relative to a control condition (a spelling game). Evans et al. [79] concluded that “there is no evidence that using this game will result in any benefit in terms of their functioning and presenting problems.” (p. 125).

Cenextra also showed early promise in a head-to-head comparison with behavioral parent training (BPT) indicating favorable feasibility, acceptability, and engagement data. Cenextra was superior to BPT for improving working memory (*d* = 1.06) and reducing objectively-assessed hyperactivity (*d* = 0.74), and equivalent to BPT for reducing parent-reported ADHD symptoms at post-treatment [90]. These benefits were largely confirmed in an RCT comparing Cenextra with an active, credible digital therapeutic control called Inhibitory Control Training (ICT; [84]). Evidence suggesting improved functional outcomes is also emerging, and includes superior improvements relative to both BPT and ICT on masked teacher perceptions of organizational skills, academic success, impulse control, and academic productivity 1–2 months after treatment ended [91, 92].

Similar to EndeavorRX and Cenextra, *Cogmed* showed considerable promise in early trials, and is arguably the most extensively studied neurocognitive training program. Results from meta-analytic reviews and RCTs, however, suggest that CogMed does not improve working memory, but rather improves select components of short-term memory (meta-analytic *d* = 0.63; [81, 86, 93, 94]. This is an important limitation for at least two reasons: First, most children with ADHD do not have deficits in short term memory (20–38% impairment rates) despite the majority having impairments in working memory (75–81%; [95–97]). Second, short-term memory abilities are not significantly associated with ADHD symptoms in most studies [81, 95], which suggests limited potential for downstream improvements in ADHD behavioral symptoms. Indeed, conclusions from multiple meta-analytic reviews suggest that benefits on ADHD behavioral symptoms from CogMed are generally limited to unblinded parent ratings [81, 93]. Notably, however, a more recent meta-analysis published by the developer of CogMed suggests significant, small benefits for reducing inattention (*d* = 0.37; [98]), though the extent to which this was driven by unblinded parent ratings was unclear.

A key benefit of digital therapeutics – and ‘software as medicine’ in general – is that they have the potential to continually adapt and improve based on real-time patient data. Thus, it is possible that interventions that are not showing the behavioral/functional benefits we had hoped for now could begin to do so in the future. Thus, we highlight some key areas for improvement based on conceptual models and the limited available literature on moderators of cognitive training efficacy for children with ADHD. First, there is a need to *maximize dosage*. In the context of digital therapeutics, ‘dosage’ refers to the quantity and quality of time spent actively engaging with the training exercises. Most existing protocols have been studied over a relatively limited time frame of 4–10 weeks of training, with a total training time of about 10–12 h across intervention protocols [83, 84, 99]. It is possible that this level of training is insufficient for producing large enough neurocognitive improvements to translate into meaningful – and statistically detectable – gains in downstream behavioral/functional outcomes, suggesting the need for more intensive/longer duration training.

An option closely related to maximizing dosage is *increasing the specificity* of the neurocognitive training target(s). Although training a variety of neurocognitive functions is appealing at face value, meta-analytic evidence indicates that such protocols produce smaller near-transfer effects than protocols that focus on a single neurocognitive training target [81]. It is presumed that the reason for this finding is that the more different cognitive ‘muscles’ that we are trying to train, the less time we can spend on any one of those ‘muscles’. Thus, it appears likely that maximizing efficacy will require separate protocols for each neurocognitive function that is impaired in ADHD, combined with a ‘personalized medicine’ approach in which each child’s neurocognitive profile is estimated at pre-treatment, and then a treatment plan is developed to target each of their identified weaknesses. We must *leverage basic science to link training targets with behavioral/functional outcomes*. Related to dosage and specificity issues is the idea of matching neurocognitive training targets with the specific outcome(s) of interest. Stated bluntly, neurocognitive training is not likely to be helpful if we are training neurocognitive abilities that are not robustly linked with the reason(s) a child presents for treatment. On the other hand, neurocognitive training protocols have great potential if they are able to produce robust improvements in their training target, *and* if that training target is robustly associated with the observable behaviors/functional outcomes we are trying to improve. A final area that shows promise for improving the efficacy of digital therapeutic interventions is augmentation: Combining them with existing treatments to (potentially) produce synergistic and/or augmentative benefits. This area of inquiry is in its infancy, and currently shows more conceptual promise than actual benefits [100].

## Future directions in improving efficacy and effectiveness

This brief review of psychosocial treatments for ADHD illustrates the robust evidence in support of these interventions. Importantly, there is no panacea or magic bullet for ADHD; the interventions reviewed herein have notable limitations and response to interventions vary. As such, there continues to be efforts at refining existing approaches and developing novel approaches to treating the complex presentation of ADHD. We highlight here what we believe are key future directions, broadly speaking, in improving the effectiveness and efficacy of treatment for ADHD. These fall under two broad areas: future directions in treatments and future directions in service delivery of these treatments.

### Future directions in treatment for ADHD

#### Moderators of treatment effects

Much of the intervention literature has focused on static factors or social addresses (terms that describe rather than explain; e.g., marital status, child age;) as factors, largely because these are measures of convenience. Efforts toward using dynamic factors have shed light on what works for whom and can further refine an intervention to increase potency. As an example, in BPT, parent-level variables (e.g., parenting stress [101]) have been shown to moderate BPT engagement and outcomes, suggesting that future refinements to BPT that more directly address parental stress may increase the potency of BPT. Such efforts should be employed across all psychosocial interventions for ADHD. Importantly, as we have discussed elsewhere [8, 102], efforts should go beyond variable-centered approaches (e.g., child age, parent stress) toward holistic, person- and/or family-centered approaches (the clustering of variables that more fully represent a child/parent/family). As an example, Dale et al. [103] employed a person-centered approach to create subgroups of families based on the intersection of multiple parent, child, and family factors to understand response to BPT for families of preschool children with ADHD. Three distinct family profiles emerged, with data suggesting differential response for families with high stress, elevated parental anxiety, and elevated parental depression. These typological approaches better reflect reality- people are more accurately reflected as a complex intersection of variables rather than just any one variable- and taking this approach may better result in a nuanced understanding of response to treatment and further inform treatment for types of people and families.

ADHD is complex and presentations vary. It is not uncommon for children and adolescents with ADHD to also have significant difficulties outside of core symptoms of ADHD (e.g., emotion dysregulation, sleep disturbances) that may moderate treatment response. As an example, ADHD is frequently associated with emotional regulation challenges, with studies suggesting that the vast majority of children with ADHD (i.e., 75%) have some symptoms of emotion dysregulation, with 25% having severe emotion dysregulation [104]. In fact, children with ADHD and severe emotion dysregulation are more likely to have complex presentations, cross-situational impairments and severe psychopathology [105]. Interestingly, there are few rigorous randomized controlled trials evaluating the effects of psychosocial interventions for emotion dysregulation in children with ADHD [106]. These and other moderators may best be evaluated through novel approaches such as individual participant data meta-analysis [107]. Addressing the needs of youth with ADHD and their families will require going beyond addressing core symptoms of ADHD. In fact, we have long argued that these types of functional impairments (e.g., academic, social functioning) should be the targets for ADHD treatment rather than ADHD symptoms [8].

#### Novel intervention targets

The presentation of ADHD at key developmental periods/tasks may pose significant challenges for affected youth and their families- necessitating novel targets of intervention. An example of this is the transition to learning to drive. Adolescents are the riskiest drivers on the roadway, overall; if an adolescent has ADHD they are significantly more at risk for negative driving outcomes including accidents, accidents that cause injury, and fatalities [108, 109]. The period of time when individuals with ADHD are learning to drive may therefore be a critical, and also opportune time to initiate intervention. A recent RCT with adolescents with ADHD [110], evaluated a specially designed computerized simulated-driving program with feedback and found reduced problematic driving as compared with a control program. During real-world driving in the year after training, the rate of collisions and near-collisions was lower in the intervention group. These efforts highlight the potential of psychosocial interventions for addressing impairments inherent with developmental transitions for youth with ADHD. Related work suggests that intervening with adolescents at the transition to middle school and high school with intensive summer “bridge” programs may be a useful approach with high levels of engagement [111]. Future work should focus on embedding treatment efforts into other developmental tasks/transitions/tasks (e.g., start of preschool or initiation of employment).

### Future directions in service delivery

ADHD is now very clearly understood to persist throughout development and into adulthood and there has rightfully been a shift toward a chronic care model [7]. Given this, attention must be given to developing integrated, consistently available and longitudinal approaches embedded in routine service systems such that children, adolescents (and even adults) with ADHD and their families can receive appropriate care. Unfortunately, availability and access to evidence-based interventions are limited. Recent studies suggest that only 31% of families of children with ADHD receive BPT [21] and just 32% receive behavioral classroom management [112]. Given this, we highlight herein issues related to increasing availability and access to evidence-based psychosocial treatment for ADHD- important goals to help close the science-to-service-gap in ADHD. More specifically, we briefly highlight efforts on (1) leveraging the existing workforce, and (2) using technology to deliver evidence-based psychosocial treatments.

#### Leveraging existing workforces

In light of mental health workforce shortages (https://data.hrsa.gov/topics/health-workforce/workforce-projections) new models of care will need to utilize and expand existing, but underdeveloped, non-professional and paraprofessional workforces [31, 32]. One example of a sustainable, scalable model of care is the Family Peer Advocate (FPA) ADHD Model [25]. FPAs are part of a national family support model of current and former parent/caregivers of children with identified mental health needs who provide a range of services, including parenting skills training, emotional support, education about mental health services, and direct advocacy [30]. FPA-services are flexibly delivered in a variety of parent-identified settings (e.g., parent’s homes, community settings) and often connect and engage parents with key service settings/providers (e.g., schools, primary care, mental health clinics), reducing the systemic barriers associated with traditional service delivery models. Moreover, FPAs have many shared experiences with the families they serve including personal experience with providing care and navigating the service system for children with mental health challenges, often within the same community as those they serve. As a result, FPA care is associated with high acceptability ratings and increased engagement [113]. This FPA Model appears to be especially effective in reaching ethnically diverse families from socioeconomically disadvantaged backgrounds [30, 113]. Emerging data suggest that FPAs can reliably and effectively deliver BPT for youth with ADHD [25], suggesting that this and other workforce (e.g., ADHD Coaches) should be leveraged in order to increase availability and access to evidence-based psychosocial interventions for ADHD.

The idea of leveraging the existing workforce also applies to school settings. The MTSS intervention framework, which embeds intervention in schools into universal, targeted, and indicated approaches is an example of a potential means of re-allocating professional time and expertise. Rather than waiting for school psychologists and special educators to get involved only when the child is considered for special education, a MTSS approach might utilize the expertise of these professionals to consult with the general education teacher on how to implement positive behavior supports for a child with ADHD. In this way, intervention is implemented quicker, in the setting where the initial impairment is identified, by existing school professionals [56, 114].

#### Use of technology

Technology-based approaches to delivering evidence-based interventions have the potential to revolutionize mental health service access and delivery across multiple mental health disorders [115]. Online self-directed BPT approaches are potentially more feasible, affordable, and acceptable, can have significant reach to include traditionally underserved populations, and are readily scalable and sustainable [116, 117]. Over 13 studies have recently been conducted demonstrating that online BPT can improve child behavioral outcomes. Importantly, a recently published trial compared Triple P Online (TPO; an evidence-based, commercially available, self-directed online BPT) to a face-to-face (F2F) therapist-delivered Triple P for preschool children with disruptive behavior problems [118]. This large randomized controlled trial found that TPO was non-inferior to F2F Triple P on observed and parent-reported child behavior, with clinically meaningful effect sizes. This study, combined with several other studies [27, 119, 120] demonstrate the effectiveness of online BPT in general and specifically for ADHD. Technology is increasingly being utilized in practice settings (e.g., ADHD Care Assistant in primary care [121]; Online Daily Report Card in schools [122]) to increase access to evidence-based psychosocial interventions. Concerted efforts and rigorous empirical investigation will be necessary to further determine the effectiveness of these approaches. Importantly, while there is high potential for such technology-delivered approaches, engagement to these formats will remain important to address [123].

## Conclusions

ADHD is a prevalent, pervasive, chronic and impairing disorder that necessitates early, integrated, continuous interventions over a child’s development. Fortunately, several psychosocial interventions are available that address key functional impairments in children and adolescents with ADHD. Given the complex presentation of ADHD, novel approaches to address both underlying pathophysiological mechanisms associated with ADHD (e.g., working memory) as well as domains closely impacted in those with ADHD (e.g., emotion regulation) and key developmental tasks (e.g., driving) are emerging areas in ADHD intervention science. Efforts to improve the efficacy of psychosocial interventions remain important as the acute benefits of these interventions do not result in normalized functioning for many youth and there remains an under-appreciation for whom these interventions are most impactful. Likely most pressing is the translation of the intervention science to improve outcomes for the millions of youth affected by ADHD, the science-to-service gap is prominent; many children who can benefit from evidence-based psychosocial interventions do not receive them. Improving access and availability of evidence-based psychosocial interventions remains critical to ensure that the significant efforts made over decades in developing and evaluating interventions for ADHD result in population-level benefits for youth with ADHD.
